# In reference to “A guideline-based preference elicitation tool to enhance shared decision-making in supervised exercise therapy for patients with intermittent claudication: a process evaluation”

**DOI:** 10.1080/07853890.2025.2581816

**Published:** 2025-11-03

**Authors:** She-Yuan Ding

**Affiliations:** School of Clinical Medicine, School of Stomatology, The Affiliated Hospital of Hangzhou Normal University, Hangzhou, China

To the Editor

Marcellis et al. evaluate a guideline-based preference-elicitation tool for shared decision-making (SDM) in supervised exercise therapy (SET) for intermittent claudication: e-learning completion 64%, use 45%, reach 38%; 39 barriers and 37 facilitators. Findings align with peripheral artery disease (PAD) guidance endorsing first-line SET and value-aligned care [1].

Clinically, my aim is to close the evidence–practice gap without sacrificing point-of-care feasibility. Two observations and three questions follow. First, strong evidence shows patient decision aids increase knowledge, value-congruent choices and participation – outcomes central to planning SET [2]. Second, when integrated into consultations, ‘encounter’ decision aids can improve patient–clinician collaboration; however, they often falter at scale because of workflow and cultural barriers – limitations the authors also observed [3]. Thus, I pose three questions. First, can adoption be accelerated by aligning implementation outcomes – acceptability, appropriateness and feasibility – with prespecified targets and by using rapid cycles of user-interface and workflow refinement? Second, which fidelity markers – for example, the point in the consultation when the tool is used and the proportion of domains completed – best predict downstream clinical behaviour (adherence to SET) and patient outcomes (walking distance and health-related quality of life (HRQoL))? Third, how can the tool be optimized for patients with lower health literacy or executive dysfunction without lengthening visits?

Despite its useful catalogue, three gaps remain. First, dose and timing of tool use are not tied to hard outcomes – functional/maximal walking distance or VascuQoL-6 change – limiting clinical inference beyond engagement [4]. Second, a therapist-only lens risks optimism bias and selective use with ‘good-fit’ patients; patient-reported data across health-literacy strata are needed. Third, align implementation metrics with a standard taxonomy (acceptability, adoption, appropriateness, feasibility, fidelity, cost, penetration, sustainability) and prespecify success thresholds to permit cross-setting, longitudinal comparison [5].

Two mechanisms could tighten causal inference and inform scale-up. Behavioural: preference elicitation around SET and lifestyle change may boost autonomy support, intrinsic motivation and adherence – mediators testable between tool use and functional gains. Physiologic: adherence to guideline-concordant SET should improve walking capacity and PAD-specific HRQoL. Use VascuQoL-6 (MID) and standardized baseline/follow-up treadmill tests to strengthen effect and value estimates [4]. Methodologically, a preregistered, cluster-randomized or stepped-wedge implementation trial embedded within Chronic CareNet could evaluate: (i) fidelity-guided implementation (e.g. EHR/portal prompts and brief scripting), (ii) equity adaptations (plain-language summaries and pictograms) and (iii) endpoints aligned with the RE-AIM and Proctor frameworks (fidelity, penetration, sustainability), assessed alongside patient outcomes and visit duration [5].

In sum, uptake was modest – adopted by a minority of therapists and used in about one-third of eligible patients – signalling room to improve workflow fit and clinician behaviour. Next steps: co-design equity-oriented UI/communication for low-literacy users; prespecify implementation outcomes with success thresholds; prospectively link fidelity/timing to SET adherence, walking distance and VascuQoL-6; and compare implementation strategies (audit-and-feedback, peer champions) in pragmatic cluster designs. Aligning this work with PAD guidance on first-line SET and decision-aid evidence should translate into clearer patient-centred benefits.

## Data Availability

Data availability is not applicable to this article as no new data were created or analysed in this study
